# Acid Dissociation Constants of the Benzimidazole Unit in the Polybenzimidazole Chain: Configuration Effects

**DOI:** 10.3390/molecules27031064

**Published:** 2022-02-04

**Authors:** Liudmil Antonov, Susumu Kawauchi, Kei Shirata

**Affiliations:** 1 Institute of Electronics, Bulgarian Academy of Sciences, 1784 Sofia, Bulgaria; 2 Central Scientific Research Laboratory, University of Chemical Technology and Metallurgy, 1756 Sofia, Bulgaria; 3 Tokyo Tech Academy for Convergence of Materials and Informatics (TAC-MI), Tokyo Institute of Technology, S6-23, 2-12-1 Ookayama, Meguro-ku, Tokyo 152-8550, Japan; 4 Department of Organic and Polymeric Materials, Tokyo Institute of Technology, 2-12-1 Ookayama, Meguro-ku, Tokyo 152-8550, Japan; keishirata@gmail.com

**Keywords:** p*K*_a_, density functional theory, benzimidazole, polymer ion, polymer electrolyte membrane fuel cells

## Abstract

The acid dissociation constant of three benzimidazoles, namely 2,2′-bibenzo[d]imidazole, 2,5′-bibenzo[d]imidazole, and 5,5′-bibenzo[d]imidazole, have been investigated by means of density functional theory calculations in gas phase and in aqueous solution. The theoretical approach was validated by the comparing of predicted and experimentally determined p*K*_a_ values in imidazole, benzimidazole, and 2-phenylbenzimidazole. From the studied compounds, 2,2′-bibenzo[d]imidazole was found to be the most acidic, which made it a valuable candidate as a material for polymer electrolyte membrane fuel cells.

## 1. Introduction

Acid dissociation constant is one of the fundamental chemical properties of the organic molecules, defining their ability not only to release or attract acidic protons, but also to interact with the environment. It is highly relevant for systems containing imidazole units, considering their dualistic nature caused by the availability of two nitrogen atoms, one of which can release a proton in a basic environment, while the other can accept a proton in an acidic environment. Not surprisingly, such materials, and especially benzimidazole and polybenzimidazole, have attracted substantial interest as metal extraction ligands, optical materials, reduction of carbon dioxide [1,2], pharmacological applications [3,4], and increasingly in polymer electrolyte membrane fuel cells [5,6,7,8,9].

Several reports have been published concerning the p*K*_a_ evaluation of imidazoles and benzimidazoles, using both experimental and computational methods. For example, Brown and Mora-Diez calculated the p*K*_a_ of protonated benzimidazoles and their derivatives using B3LYP/6-31+G(d,p) level of theory with the polarized continuum model (PCM), in order to develop a procedure for evaluating p*K*_a_ of protonated benzimidazoles [10,11]. Zhang calculated nearly 500 organic bases, including imidazole and benzimidazole, using conductor-like screening model (COSMO) and empirical correction to establish a protocol to calculate p*K*_a_ for organic bases [12]. A p*K*_a_ value of 7.0 for protonated imidazole has been reported from an experimental measurement [13], while theoretical calculations provided values of 6.8–7.1 [12,13,14,15,16]. For protonated benzimidazoles, the p*K*_a_ value is found to be 5.4–5.5 from experimental measurements [17,18] and 5.6–5.8 from computational calculations [15,16,19].

In the computational studies, the p*K*_a_ of hypothetical molecule AH dissociating into A^−^ and H^+^ is determined using its relationship with the Gibbs free energy of deprotonation in aqueous phase ($\Delta G_{\mathrm{dep}\left(\mathrm{aq}\right)}^{*}$) as follows:

(1) $$pK_{a}=\frac{{\Delta G}_{\mathrm{dep}\left(\mathrm{aq}\right)}^{*}}{RT\ln10}$$

**Scheme 1:**
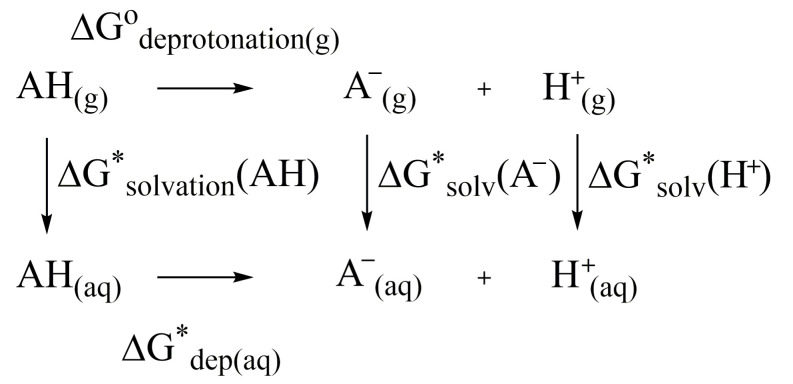
Thermodynamic cycle used to evaluate acid dissociation constants ($\Delta G_{\mathrm{deprotonation}\left(g\right)}^{o}$—free energy of deprotonation in the gaseous phase; $\Delta G_{\mathrm{solvation}}^{*}$ —free energy of solvation of the particular species).

$\Delta G_{\mathrm{dep}\left(\mathrm{aq}\right)}^{*}$ can be evaluated using thermodynamic cycle represented in Scheme 1:

Therefore, $\Delta G_{\mathrm{dep}\left(\mathrm{aq}\right)}^{*}$ may be evaluated using the following equation:

(2) $$\Delta G_{\mathrm{dep}\left(\mathrm{aq}\right)}^{*}=G^{*}(A_{\left(\mathrm{aq}\right)}^{-})+G^{*}(H_{\left(\mathrm{aq}\right)}^{+})-G^{*}({\mathrm{AH}}_{\left(\mathrm{aq}\right)})\;=(G^{o}\left(A_{\left(g\right)}^{-}\right)+{\Delta G}^{1\mathrm{atm}\to 1M}+{\Delta G}_{\mathrm{solv}}^{*}\left(A^{-}\right))+(G^{o}\left(H_{\left(g\right)}^{+}\right)+{\Delta G}^{1\mathrm{atm}\to 1M}+{\Delta G}_{\mathrm{solv}}^{*}\left(H^{+}\right))-\left((G^{o}\left({\mathrm{AH}}_{\left(g\right)}\right)+{\Delta G}^{1\mathrm{atm}\to 1M}+{\Delta G}_{\mathrm{solv}}^{*}\left(\mathrm{AH}\right)\right)$$

where ${\Delta G}^{1\mathrm{atm}\to 1M}$ is the free energy of compression from 1 atm to 1 M (1.89 kcal/mol). This cycle is not only applicable to single proton dissociation of (AH/A^−^), but also to any one step deprotonation between charged states.

It should be stressed that the acid dissociation constant is highly sensitive to the value of $\Delta G_{\mathrm{dep}\left(\mathrm{aq}\right)}^{*}$, since a change in only 6 kJ/mol can make a difference of 1 unit in the p*K*_a_ value. Therefore, efforts have been made to rise its precision either through improvement of the solvation Gibbs free energy of the proton [20,21] or by adding an empirical correction to the p*K*_a_ value itself [11,22,23]. While the latter tends to yield extremely accurate values, it is based on a large set of experimental values of structurally similar molecules and its applicability is limited only for similar molecules and/or exactly the same level of theory (functionals and basis sets). The Complete Basis Set method (CBS-QB3) of Petersson and coworkers [24] is often used for an accurate prediction of gas phase energies [25,26,27], but the heavy reliance on computational resources restricts this method to small molecules. Perhaps the most logical approach is to improve the accuracy of the free energy of solvation evaluation. Broadly, two different approaches have been attempted, namely the improvement of the solvation model or by using explicit solvent molecules. Traditionally, PCM [28] or conductor-like polarized continuum model (CPCM) [29,30] can be chosen, but in the more recent years, solvation models such as the universal solvation model based on solute electron density (SMD) [31] or COSMO and its variants [32,33] are seeing an increasing popularity. Recently, some theoretical studies suggest that SMD can be a suitable solvation model for the calculation of solvated states [26,27,34]. Another option, which is potentially more realistic when specific solute-solvent interactions are possible, is to use single explicit water molecule(s) as a part of the calculation, known as the cluster continuum approach. While this has been shown to improve the values of solvation free energies and p*K*_a_ [35,36,37], it is not trivial to determine the correct water-solute interaction sites and number of water molecules, in addition to their position and orientation [26].

Considering that polybenzimidazole collects an increasing amount of attention as a candidate material for polymer electrolyte membrane (PEM) fuel cells, the study of p*K*_a_ of this material has an increasing importance in both practical and fundamental functions. Polybenzimidazole doped with phosphoric acid is used as PEM material in high temperature PEM fuel cell. In a previous work, the interaction between phosphoric acid and bibenzimidazoles of three different connectivity has been studied [38], focusing on the interacting structure and energetics and showing that close proximity of imidazole rings offered by 2,2′-bibenzo[d]imidazole produces strong interaction with phosphoric acid.

**Scheme 2:**
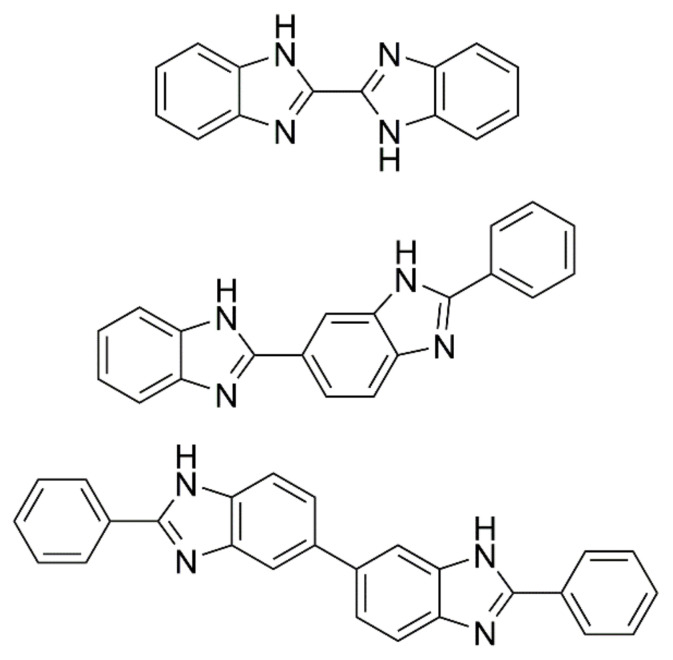
Structural diagram of 2,2′-bibenzo[d]imidazole (**2,2′-BBIm**, top), 2′-phenyl-2,5′-bibenzo[d]imidazole (**2,5′-BBIm**, middle), 2,2′-diphenyl-5,5′-bibenzo[d]imidazole (**5,5′-BBIm**, bottom).

In current study, we aim to build knowledge on how well the benzimidazole accepts proton by calculating p*K*_a_ of the protonated structures of 2,2′-bibenzo[d]imidazole, 2,5′-bibenzo[d]imidazole and 5,5′-bibenzo[d]imidazole (resp. **2,2′-BBIm**, **2,5′-BBIm** and **5,5′-BBIm** in Scheme 2). Imidazole rings are terminated by phenyl group where needed to take account of the terminal group effect. In the interest of complete study of the protonation/deprotonation process, p*K*_a_ of deprotonated structures were also calculated to theoretically predict how the molecule would act in basic environment. In our best knowledge there is no such detailed study, up to now, on the protonation processes in bibenzimidazoles.

## 2. Results and Discussion

**Figure 1:**
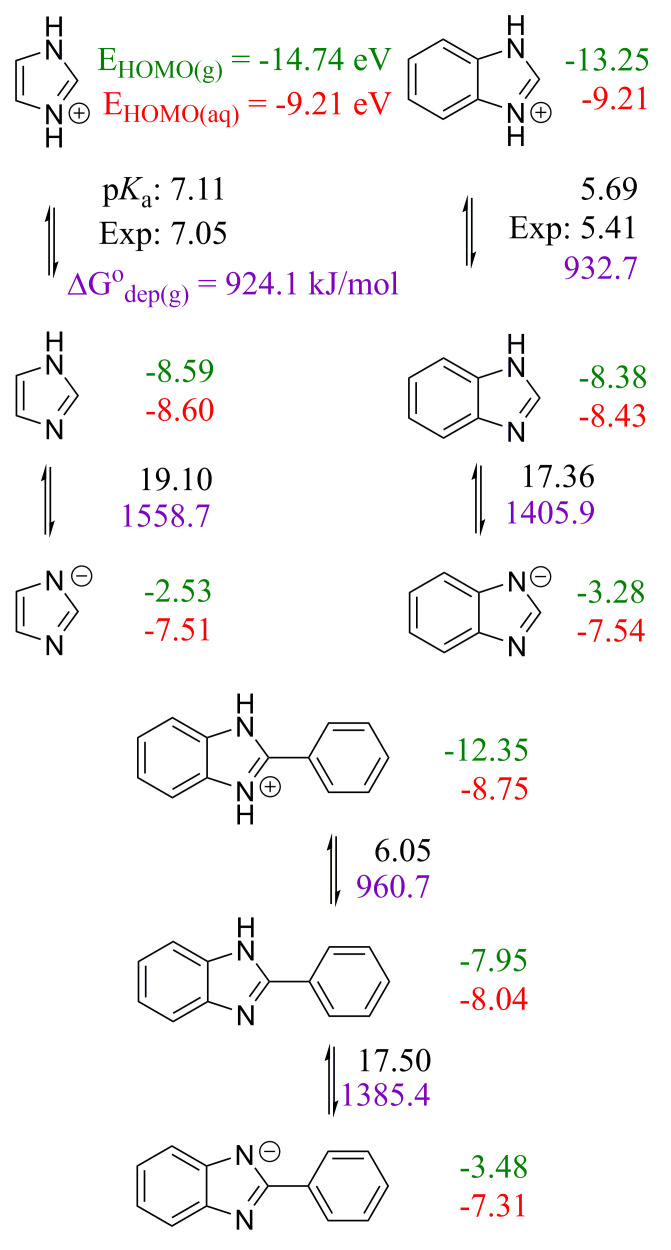
p*K*_a_ (black) and free energy of deprotonation in gas phase (purple) between structures of protonated (deprotonated) state of imidazole, benzimidazole, and 2-phenylbenzimidazole. The energy of HOMO in gas phase (green) and in aqueous phase (red) of each structure are also given. All calculated at the ωB97X-D/6-311+G(d,p) level of theory.

The p*K*_a_ values and energy of HOMO in the gas phase and aqueous phase were calculated for imidazole, benzimidazole, and 2-phenylbenzimidazole, with the results shown in Figure 1. All HOMO energy levels are in the negative range, indicating that all structures, especially anions, are valid structures that p*K*_a_ can be measured with. The energy level of HOMO decreases with solvation for neutral and deprotonated species, indicating stabilization with solvation. However, it increases for protonated species. p*K*_a_^+^ values of imidazole (experiment: 7.05, calculated: 7.11) and benzimidazole (experiment: 5.41, calculated: 5.69) agrees very well with the experiment. These p*K*_a_^+^ values show that benzimidazole is comparatively more acidic compared to imidazole. The effect of the addition of phenyl group at 2-position is assessed, to see if the addition of phenyl group may stabilize the charged species. In terms of p*K*_a_^+^ and p*K*_a_ value, 2-phenylbenzimidazole (**2-PhBIm**) is more basic, but on a very limited scale. Table 1 shows the bond length and dihedral angle between the benzimidazole and phenyl group of **2-PhBIm** and its protonated (**2-PhBIm^+^**) and deprotonated (**2-PhBIm^−^**) counterparts. Both the gas phase and aqueous phase data are given. Bond length increases in the deprotonated molecule, leading to a difference between the cationic and anionic species of 0.02 Å. The phase has a large effect on the dihedral angle. In the gas phase, dihedral angle decreases as **2-PhBIm** becomes deprotonated, with the anion being coplanar (protonated: 33.90°, neutral: 17.21°, deprotonated: 0.01°). However, this effect is lightened in solution, with both **2-PhBIm^+^** and **2PhBIm^−^**, showing values closer to the neutral species (protonated: 24.19°, neutral: 20.73°, deprotonated: 16.35°). An increase in the dihedral angle from deprotonated species to protonated species can be explained by an increase in the steric repulsion between hydrogen atoms on the imidazole ring and hydrogen atom on the phenyl group. The deprotonated species in solution should also have a dihedral angle of 0°, but rather, the gas-phase calculations probably overemphasize π-conjugation, resulting in a planar structure.

### 2.1. **2,5′-BBIm**

**Figure 2:**
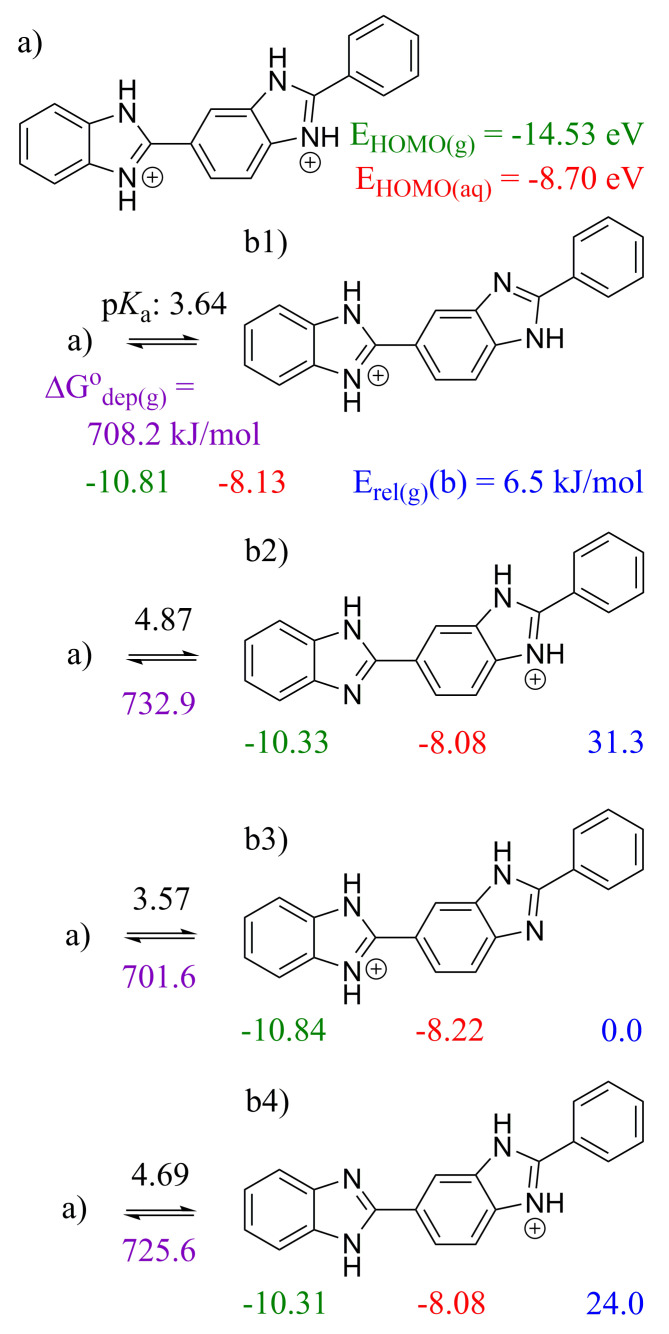
p*K*_a_ (black) and free energy of deprotonation in the gas phase (purple) between structures of twice (**a**) and once (**b**) protonated **2,5′-BBIm**. The energy of HOMO in the gas phase (green) and aqueous phase (red) is shown. The relative energy between different configuration and conformations within structure **b** is also shown. All calculated at the ωB97X-D/6-311+G(d,p) level of theory.

**Figure 3:**
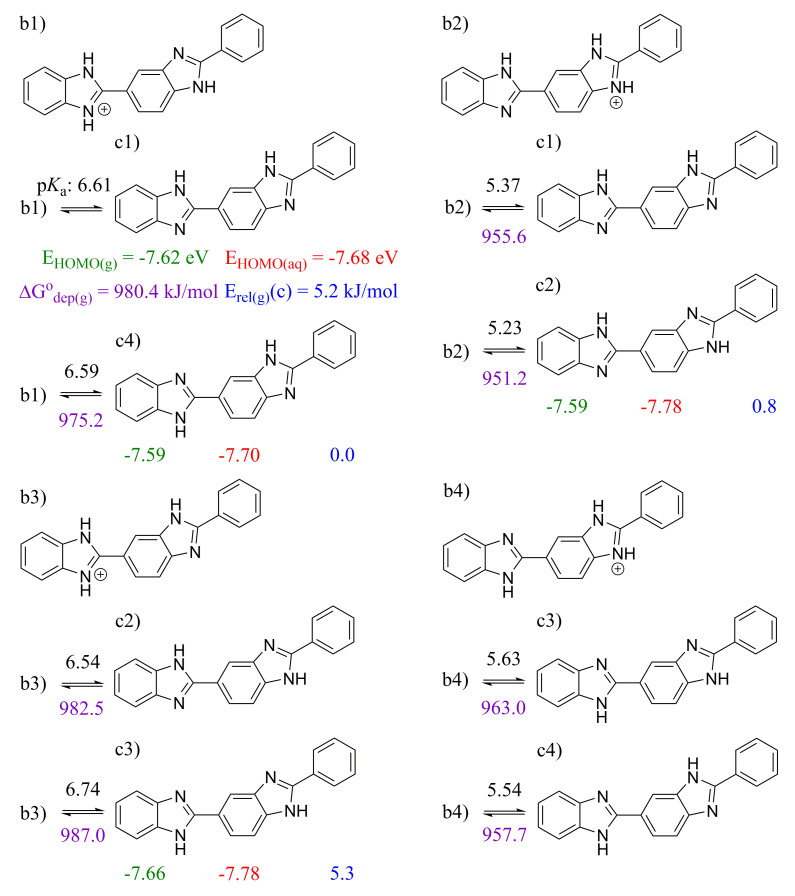
p*K*_a_ (black) and free energy of deprotonation in the gas phase (purple) between structures of once protonated (**b**) and neutral (**c**) **2,5′-BBIm**. The energy of HOMO in the gas phase (green) and aqueous phase (red) is shown, unless it has been stated in a previous figure. The relative energy between different configuration and conformations within structure **c** is also shown. All calculated at the ωB97X-D/6-311+G(d,p) level of theory.

**Figure 4:**
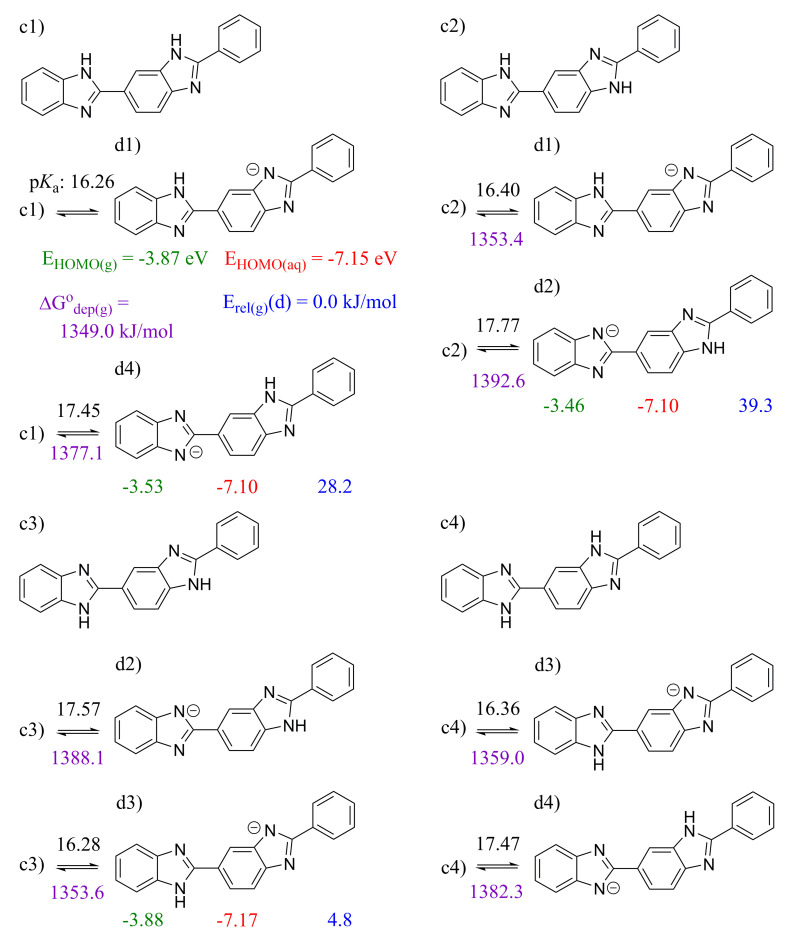
p*K*_a_ (black) and free energy of deprotonation in the gas phase (purple) between structures of the neutral (**c**) and single deprotonated (**d**) **2,5′-BBIm**. The energy of HOMO in the gas phase (green) and aqueous phase (red) is shown, unless it has been stated in a previous figure. The relative energy between different configuration and conformations within structure **d** is also shown. All calculated at the ωB97X-D/6-311+G(d,p) level of theory.

**Figure 5:**
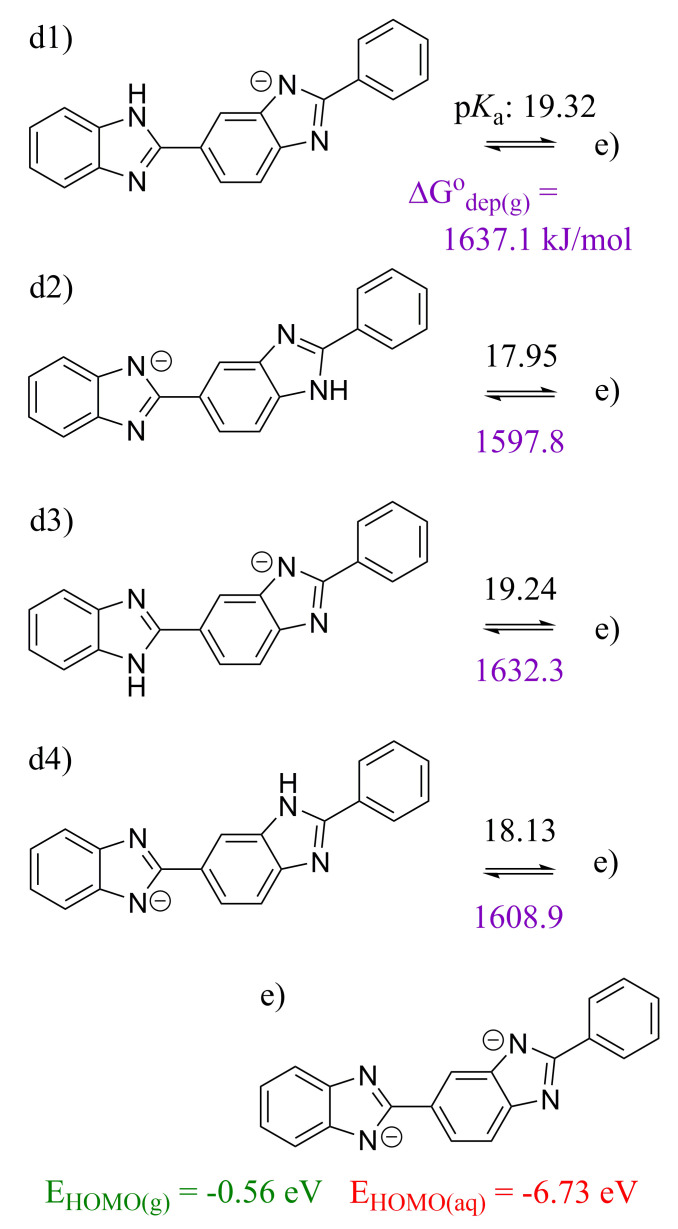
p*K*_a_ (black) and free energy of deprotonation in the gas phase (purple) between structures of once (**d**) and twice (**e**) deprotonated **2,5′-BBIm**. The energy of HOMO in the gas phase (green) and aqueous phase (red) is shown, unless it has been stated in a previous figure. All calculated at the ωB97X-D/6-311G(d,p) level of theory.

In a similar manner, the p*K*_a_ values and energy of HOMO in gas phase and aqueous phase were calculated for **2,5′-BBIm**. The results are shown in Figure 2, Figure 3, Figure 4 and Figure 5, where each figure represents the p*K*_a_ values between one protonated (deprotonated) state and another. The structures of **2,5′-BBIm** are labeled according to their protonated state. Structure **a** is **2,5′-BBIm** protonated twice, and there is only one conformation available for a structure of bibenzimidazole that is protonated twice. Structure **b** is **2,5′-BBIm** protonated once. Since there are two imidazole rings, there are potentially four nitrogen atoms that can be protonated. Hence, there are three different configurations (**b1**, **b2**, and **b3**), one of which has two different conformations (**b2** and **b4**). These are labeled as **b1**, **b2**, **b3**, and **b4**. There are two different conformations of two different configurations for neutral species (**c1** and **c4**, **c2** and **c3** are same configurations). For structures of **2,5′-BBIm** deprotonated once (**d1**–**d4**), there are three different configurations (**d1**, **d2,** and **d4**) of which one of them has two different conformations (**d1** and **d3**). Only one structure exists for **2,5′-BBIm** deprotonated twice, and this is labeled as **e**. Figure 2 shows the p*K*_a_^2+^ values between structure **a** and structure **b**. It evident that the p*K*_a_^2+^ varies depend on the location of the proton. p*K*_a_^2+^ of the structures with the second benzimidazole deprotonated (**b1** and **b3**) is approximately 3.6, whereas those with terminal benzimidazole deprotonated (**b2** and **b4**) is approximately 4.8. This is a result of the lower structural energy (over 15 kJ/mol) in **b1** and **b3** when compared to **b2** and **b4**, which reflects to both gas phase relative energy and gas phase free energy of deprotonation (minimum difference in the relative energy of 17.5 kJ/mol). As shown, the results indicate clear preference of deprotonation location.

The same calculations were repeated with structure **c** to evaluate values of p*K*_a_^+^, and the results are shown on Figure 3. The clear differences in **b** structures affected the values of p*K*_a_^+^. p*K*_a_^+^ of structures **b1** and **b3** showed values of approximately 6.6, whereas those of **b2** and **b4** showed range of p*K*_a_^+^ between 5.2–5.7. 

Figure 4 shows p*K*_a_^−^ values; Figure 5 shows p*K*_a_^2−^ of **2,5′-BBIm**. HOMO energy verifies the validity of p*K*_a_^−^ and p*K*_a_^2−^ calculation. p*K*_a_^−^ and p*K*_a_^2−^ values suggest that the deprotonation of **c** is unlikely to occur under typical circumstances, possessing the value of approximately 16–18 for p*K*_a_^−^ and 18–19 for p*K*_a_^2−^. However, it is worthwhile to note, since there is bias on which repeating unit is more likely to be found in a singly deprotonated state, in the same manner as structure **b**. The relative energy of **d** shows that deprotonation is more likely to occur on the non-terminal benzimidazole (**d1** and **d3**) than the terminal benzimidazole (**d2** and **d4**). In summary, there is energetic bias for terminal benzimidazole to become benzimidazolium, whereas there is energetic bias for the non-terminal benzimidazole to become benzimidazolate.

Table 2 shows the bond length and dihedral angle between the two benzimidazole repeating units of **2,5′-BBIm** and its ionic counterparts. For the neutral species **c**, there are no differences in the bond length (1.468–1469 Å) between the repeating units, but the gas phase dihedral angle differs between structures **c1**, **c3** (22°) and **c2**, and **c4** (16–17°). The difference between these structures depends on the position of the hydrogens (on the same side or different side of the molecule), and this small difference reflects the relative energies of structure **c**. As demonstrated in the discussion of p*K*_a_^+^ and p*K*_a_^−^ above, the structures of **b** and **d** differ depending on which benzimidazole is protonated (deprotonated). In the case of **b**, gas phase structures of **b1** and **b3** displays shorter bond length by approximately 0.02 Å, and wider dihedral angle at 30°, compared to 16° and 8° of **b2** and **b4**, respectively. The bond length of **b1** and **b3** shows contraction from **c**, though there is little change for **b2** and **b4**. In the aqueous phase, the bond length difference is reduced to 0.015 Å, and it is found that there is little difference in the dihedral angle of these structures. In the cases of **d**, the gas phase structures of **d1** and **d3** shows a shorter bond length than **d2** and **d4** by approximately 0.015 Å. Compared to **c**, the bond length of **d1** and **d3** contracted by 0.01 Å, whereas the bond lengths of **d2** and **d4** elongated by 0.005–0.009 Å. The dihedral angle of **d2** and **d4** shows that these structures are almost coplanar, whereas the dihedral angle of **d1** and **d3** are 13.38° and 19.12°, respectively. In the aqueous phase, there is little difference in the bond length when compared to the gas phase, but unlike structure **b**, there still remains variation of the dihedral angle between **d1**, **d3** and **d2**, and **d4,** though the differences are significantly lower of approximately 2.5–9°. There is an effect of charge repulsion between the two protonated (deprotonated) repeating units for twice protonated (deprotonated) structures **a** and **e**. For structure **a**, bond length does not differ, with respect to **d2** and **d4** or **c**, and increases with respect to **b1** and **b3** by approximately 0.02 Å. The dihedral angle increases to 43°. For structure **e**, the bond length increases to 1.483 Å, approximately 0.014 Å greater than structure **c**. It is coplanar in the gas phase but shows a dihedral angle of 0.9 Å in the aqueous phase.

### 2.2. **2,2′-BBIm**

**Figure 6:**
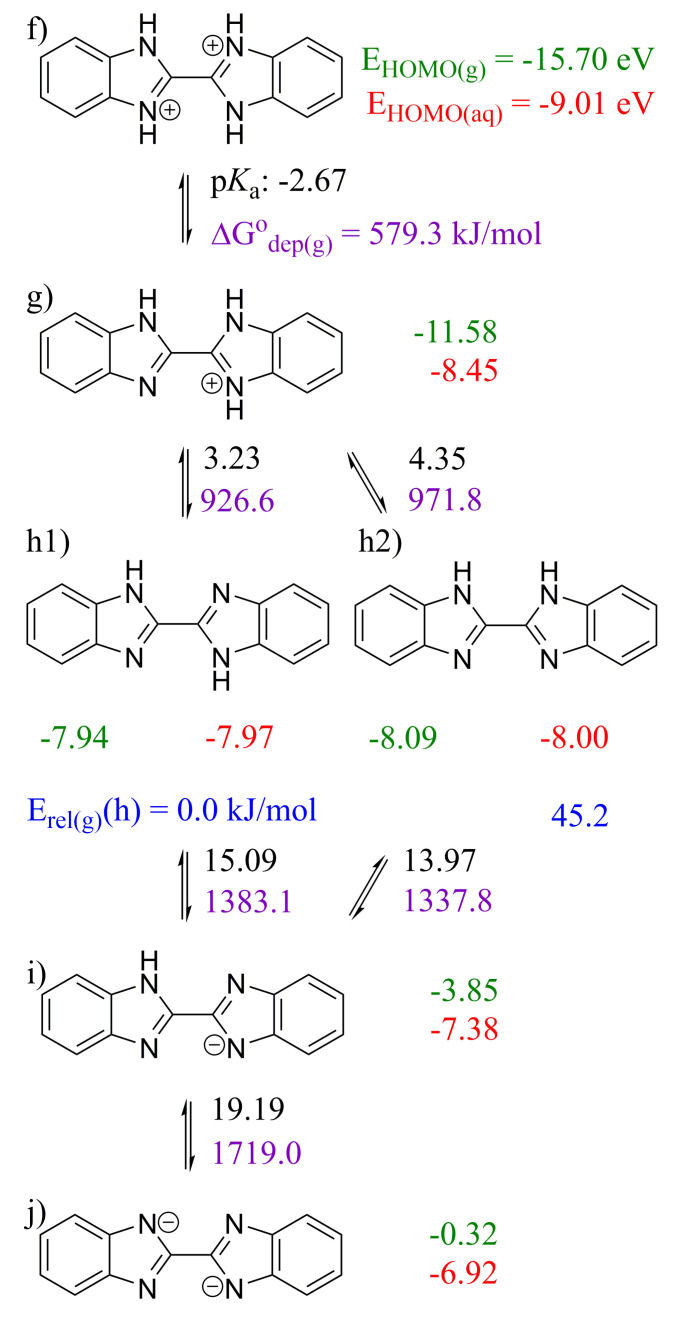
p*K*_a_ (black) and free energy of deprotonation in the gas phase (purple) between structures of **2,2′-BBIm** at various stages of protonation (deprotonation). The energy of HOMO in the gas phase (green) and aqueous phase (red) is shown. The relative energy between different configuration and conformations within the structure **h** is also shown. All calculated at the ωB97X-D/6-311+G(d,p) level of theory.

**2,2′-BBIm** has an increased degree of symmetry compared to **2,5′-BBIm**, and hence a reduced conformation variation. Possible protonated, neutral, and deprotonated structures are shown in Figure 6. HOMO energies suggest that all structures exist. The obtained energies show deprotonated structures are stabilized with solvation, while protonated structures are destabilized. For the protonated (deprotonated) structures (protonated: **f–g**, deprotonated: **i–j**), only single structure exists. There are two conformations (**h1**, **h2**) for the neutral structures, based on the rotation of the bond between two repeating units, which allows the hydrogen atoms on the imidazole ring to be on a different or the same molecular side. p*K*_a_^2+^ (−2.67), p*K*_a_^−^ (15.09, 13.97), and p*K*_a_^2−^ (19.19) suggest that **f**, **i,** and **j** are unlikely to exist under typical circumstances. On one side, this is similar to **2,5′-BBIm**, which was unlikely to yield deprotonated structures under typical circumstances with p*K*_a_^−^ value of over 16. Alternatively, there is a large difference in p*K*_a_^+^ and especially p*K*_a_^2+^ value in **2,5′-BBIm**. p*K*_a_^+^ value of **2,2′-BBIm** is 3.23 or 4.35 depending on the starting structure, which would be feasible under strong acidic conditions, but different from **2,5′-BBIm**, which had a p*K*_a_^+^ value of approximately 6. p*K*_a_^2+^ of **2,5′-BBIm** is around 4, which is a large difference from p*K*_a_^2+^ of **2,2′-BBIm** at −2.67. For the neutral species, the conformation with the hydrogen atoms on either side of the molecule (**h1**) is more stable than the conformation with hydrogen atoms on the same side (**h2**) by 45.2 kJ/mol, which reflects in the p*K*_a_^+^ and p*K*_a_^−^ values. For **h1**, p*K*_a_^+^ is 3.23 and p*K*_a_^−^ is 15.09. Whereas for **h2**, p*K*_a_^+^ is 4.35 and p*K*_a_^−^ is 13.97. This suggests that **h1** is less prone to both accepting and releasing a proton.

Structural comparison of different protonation states of **2,2′-BBIm** is made in Table 3. There is a clear difference between bond length and dihedral angle of the neutral species (**h1**, **h2**) due to the steric repulsion between the hydrogen atoms in the imidazole ring: **h1** is coplanar, whereas for **h2** it increases to 36°. The bond length of **h1** is shorter than **h2** by 0.012 Å. In the aqueous phase, the difference in bond length decreases to 0.007 Å with a reduction of the dihedral angle of **h2** to 19°. No major structural changes are seen for **h1**. For protonated species **g**, the bond length decreases by 0.01 to 1.441 Å. Interestingly, in the gas phase it is also coplanar, even though steric repulsion should exist between the hydrogen atoms on the imidazole ring. It may be possible that there is a small charge transfer from neutral benzimidazole to protonated benzimidazole, strengthening the interaction of π-orbitals and resulting in a planar structure. In the aqueous phase, there is slight deviation from coplanar structure of 5° and slight increase in bond length by 0.006 Å. As was the case with **2,5′-BBIm**, the bond length between the two benzimidazoles in twice protonated structure (**f**) increases by 0.016 Å and the dihedral angle increases to 50°. Since both benzimidazoles are equally positively charged and no charge transfer would occur, there would be compound effect from repulsion between both two positively charged benzimidazoles and steric repulsion between two pairs of hydrogen atoms. In the aqueous phase, such repulsion is greatly lightened, shortening the bond length by 0.007 Å and reducing the dihedral angle to 8°. For the single deprotonated species **i**, the gas phase structure is a coplanar structure with bond length of 1.462 Å. The structure does not change greatly in the aqueous phase, which again suggests an interaction between the neutral and negatively charged benzimidazole units through π-orbital. For twice deprotonated structure **j**, the bond length between the two benzimidazole units greatly increases to 1.486 Å as does the dihedral angle to 35°. This is likely due to the repulsion between the two benzimidazole and the π-orbitals on the imidazole ring.

### 2.3. **5,5′-BBIm**

**Figure 7:**
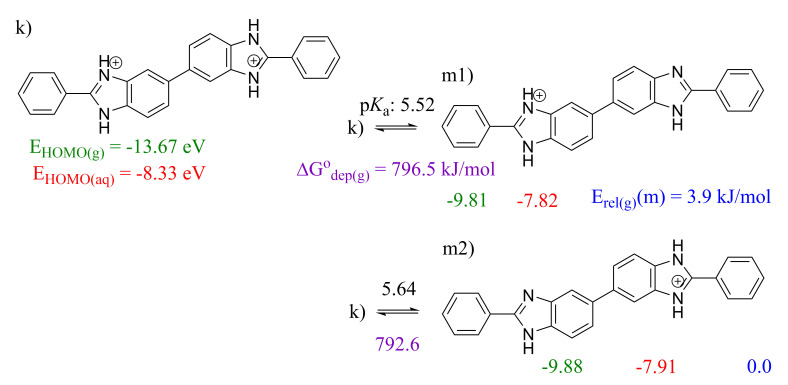
p*K*_a_ (black) and free energy of deprotonation in the gas phase (purple) between structures of twice (**k**) protonated and once (**m**) protonated **anti-5,5′-BBIm**. The energy of HOMO in the gas phase (green) and aqueous phase (red) is shown. The relative energy between different configuration and conformations within the structure **m** is also shown. All calculated at the ωB97X-D/6-311+G(d,p) level of theory.

**Figure 8:**
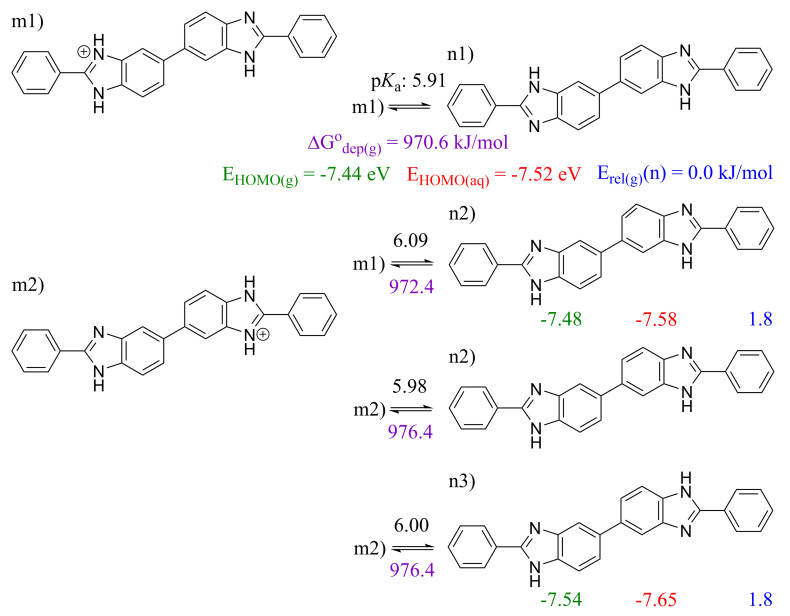
p*K*_a_ (black) and free energy of deprotonation in the gas phase (purple) between structures of once (**m**) protonated and neutral (**n**) anti-**5,5′-BBIm**. The energy of HOMO in the gas phase (green) and aqueous phase (red) is shown, unless it has been stated in previous figures. The relative energy between different configuration and conformations within the structure **n** is also shown. All calculated at the ωB97X-D/6-311+G(d,p) level of theory.

**Figure 9:**
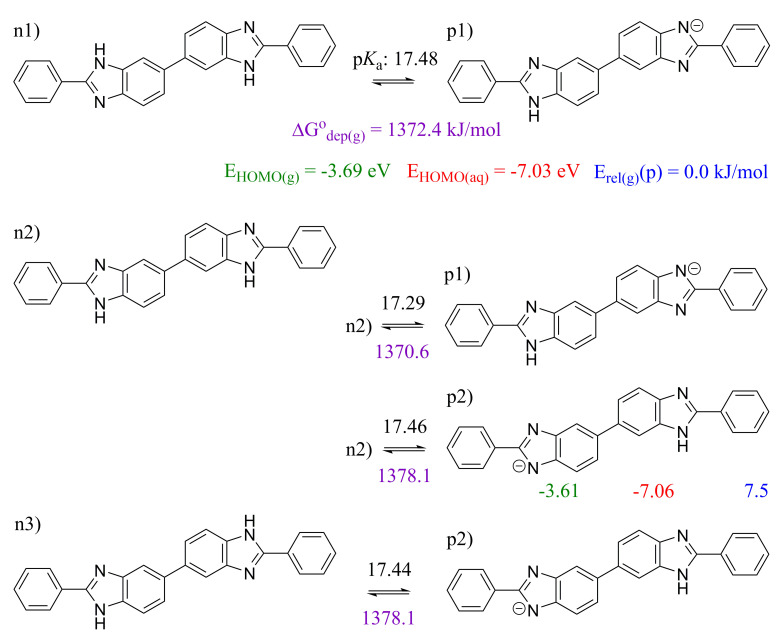
p*K*_a_ (black) and free energy of deprotonation in the gas phase (purple) between structures of neutral (**n**) and once deprotonated (**p**) anti-**5,5′-BBIm**. The energy of HOMO in the gas phase (green) and aqueous phase (red) is shown, unless it has been stated in previous figures. The relative energy between different configuration and conformations within the structure **p** is also shown. All calculated at the ωB97X-D/6-311+G(d,p) level of theory.

**Figure 10:**
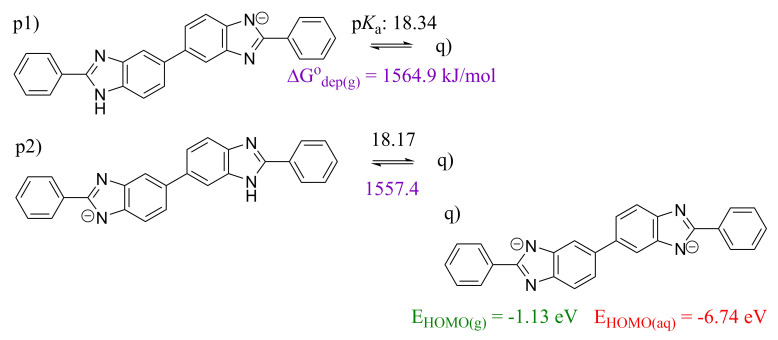
p*K*_a_ (black) and free energy of deprotonation in the gas phase (purple) between structures of once (**p**) and twice (**q**) deprotonated anti-**5,5′-BBIm**. The energy of HOMO in the gas phase (green) and aqueous phase (red) is shown, unless it has been stated in previous figures. All calculated at the ωB97X-D/6-311+G(d,p) level of theory.

**Figure 11:**
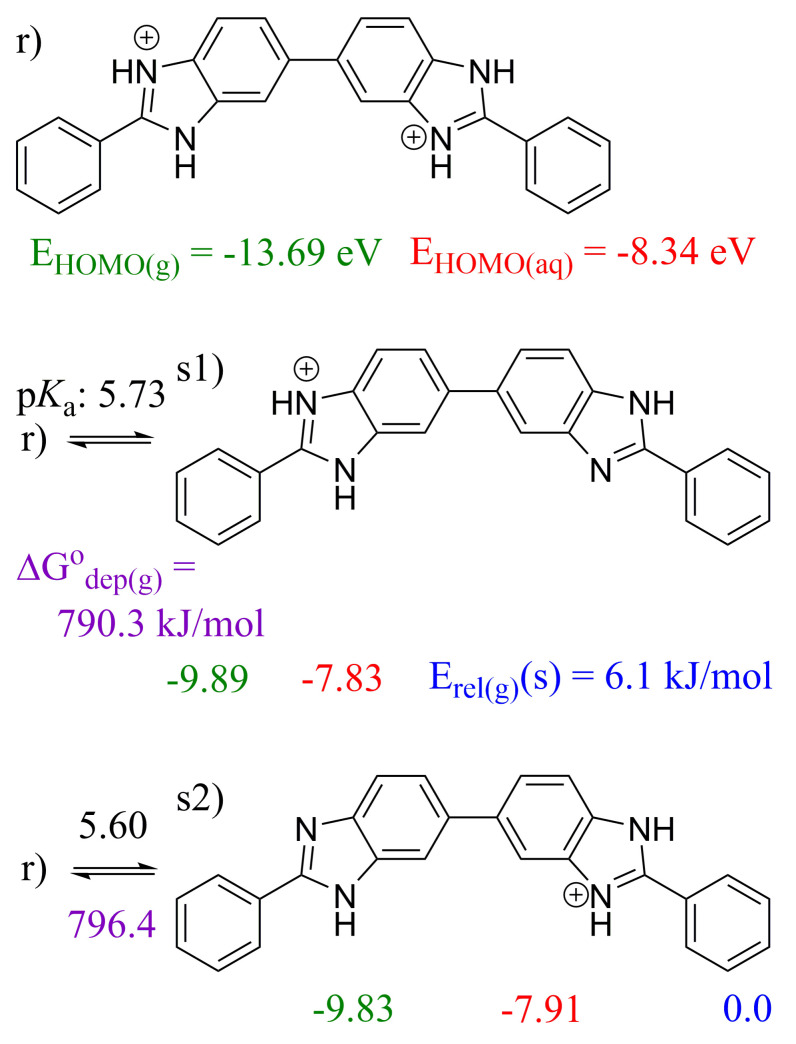
p*K*_a_ (black) and free energy of deprotonation in the gas phase (purple) between structures of ^®^ce (**r**) and once (**s**) protonated **5,5′-BBIm**. The energy of HOMO in the gas phase (green) and aqueous phase (red) is shown. The relative energy between different configuration and conformations within the structure **s** is also shown. All calculated at the ωB97X-D/6-311+G(d,p) level of theory.

**Figure 12:**
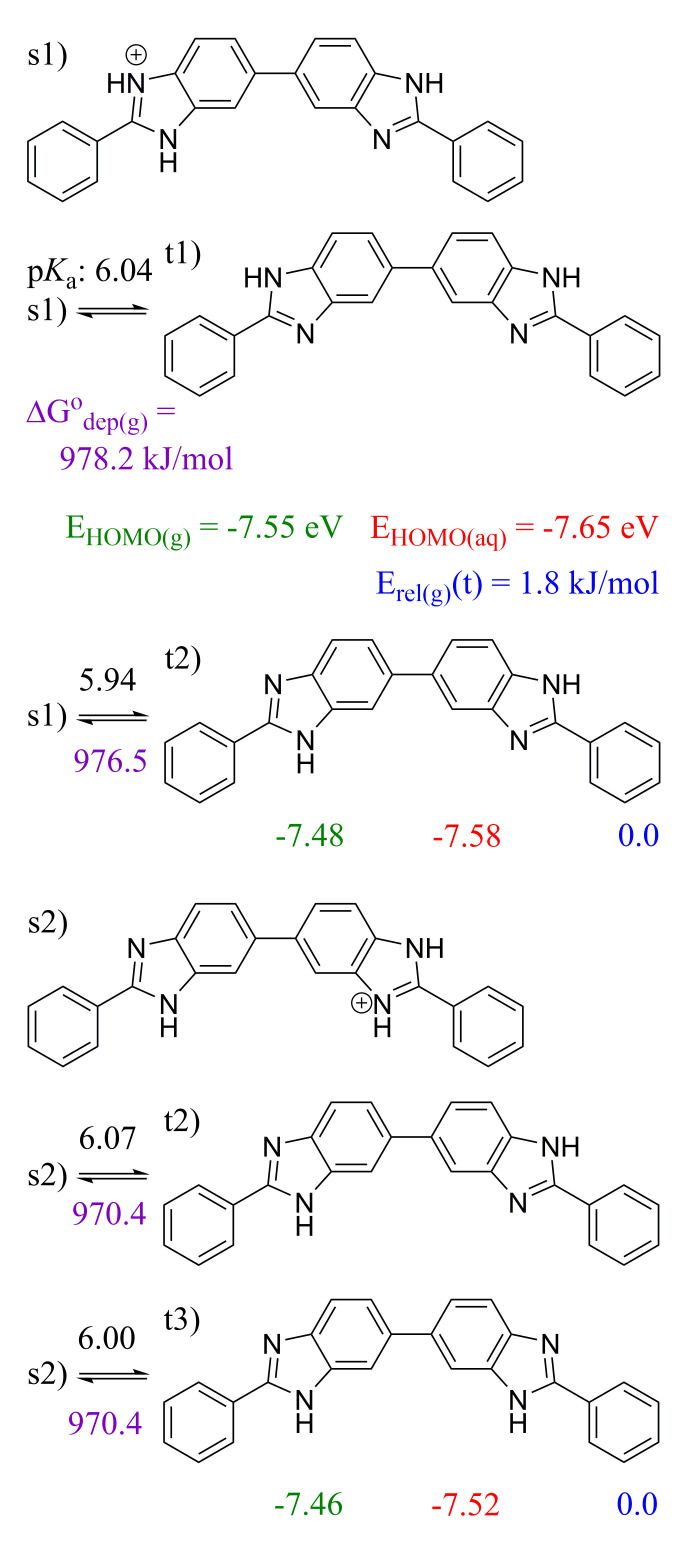
p*K*_a_ (black) and free energy of deprotonation in the gas phase (purple) between structures of once protonated (**s**) and neutral (**t**) 5,5′-BBIm. The energy of HOMO in the gas phase (green) and aqueous phase (red) is shown, unless it has been stated in previous figures. The relative energy between different configuration and conformations within the structure **t** is also shown. All calculated at the ωB97X-D/6-311+G(d,p) level of theory.

**Figure 13:**
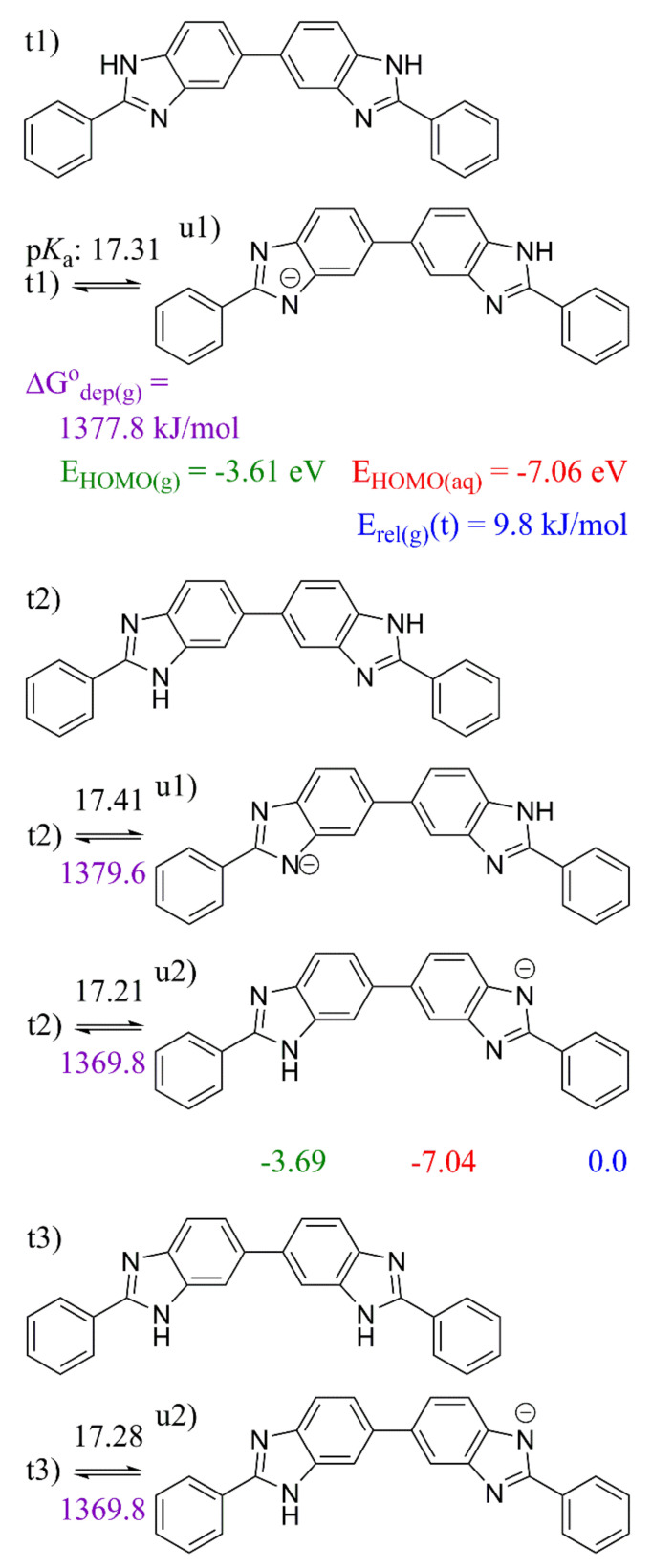
p*K*_a_ (black) and free energy of deprotonation in the gas phase (purple) between structures of neutral (**t**) and once deprotonated (**u**) **5,5′-BBIm**. The energy of HOMO in the gas phase (green) and aqueous phase (red) is shown, unless it has been stated in previous figures. The relative energy between different configuration and conformations within the structure **u** is also shown. All calculated at the ωB97X-D/6-311+G(d,p) level of theory.

**Figure 14:**
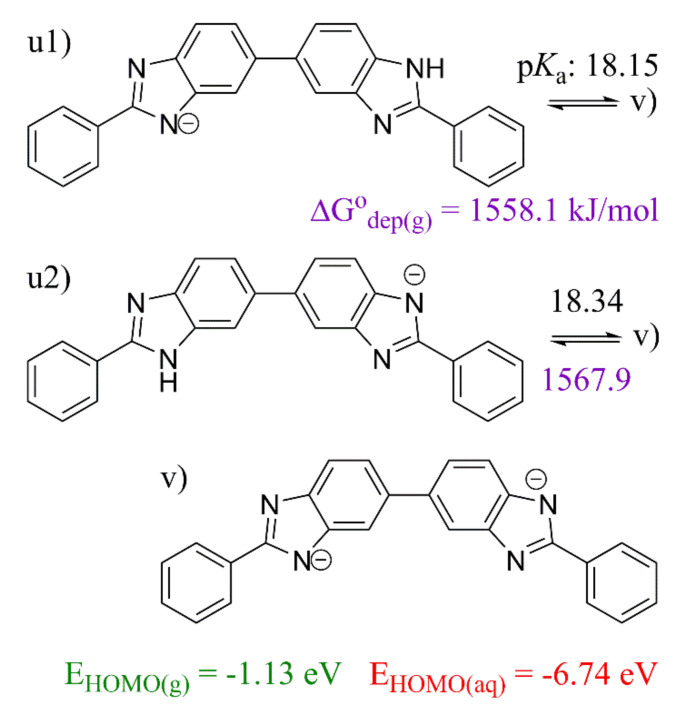
p*K*_a_ (black) and free energy of deprotonation in the gas phase (purple) between structures of once (**u**) and twice (**v**) deprotonated **5,5′-BBIm**. The energy of HOMO in the gas phase (green) and aqueous phase (red) is shown, unless it has been stated in previous figures. All calculated at the ωB97X-D/6-311+G(d,p) level of theory.

Broadly, **5,5-BBIm** may be split into two conformations. The dihedral angle of 5,5′ position may be rotated so that 2-phenyl and 2′-phenyl bonds face approximately opposite from each other (Figure 7, Figure 8, Figure 9 and Figure 10), or at an angle to each other (Figure 11, Figure 12, Figure 13 and Figure 14). Therefore, these two conformations are discussed as two separate molecules, where the former will be referred to as anti-**5,5′-BBIm** and the latter will be referred to as syn-**5,5′-BBIm**. For both, there is one twice protonated structure (**k** and **r**), two once protonated structures (**m1**, **m2** and **s1**, **s2**), three neutral structures (**n1**, **n2**, **n3** and **t1**, **t2**, **t3**), two once deprotonated structures (**p1**, **p2** and **u1**, **u2**), and one twice deprotonated structure (**q** and **v**).

Figure 7 shows p*K*_a_^2+^ of anti-**5,5′-BBIm**. There are two possible structures for single protonated structure (**m1**, **m2**). However, unlike **2,5′-BBIm**, there is no large difference between two p*K*_a_^2+^ values, since unlike **2,5′-BBIm**, there is little energetic difference between **m1** and **m2**. This suggests that the charged region of the protonated benzimidazole is hardly affected by the other benzimidazole, and they can be considered as separate units. The value of p*K*_a_^2+^ is approximately 5.5, whereas the value of p*K*_a_^+^ is approximately 6.0 (Figure 8). There is little variation in the p*K*_a_^+^ values, as the relative energy between the different **n** structures is minor. The difference between p*K*_a_^2+^ and p*K*_a_^+^ is miniscule, considering that p*K*_a_^2+^ is p*K*_a_ between twice protonated molecule and single protonated molecule, whereas p*K*_a_^+^ is p*K*_a_ between once protonated molecule and neutral molecule. p*K*_a_^+^ values of anti-**5,5′BBIm** does not differ greatly between them and are very similar to p*K*_a_^+^ of **2PhBIm**. All of these facts support that these two benzimidazole units can be considered as separate units and biphenyl moiety effectively acts as a “wall” preventing interaction between them. 

Figure 9 and Figure 10 show p*K*_a_^−^ and p*K*_a_^2−^ of anti-**5,5′-BBIm**. The HOMO energy verifies all anionic structures, though p*K*_a_ values of over 17 suggest these are unlikely to form in typical circumstances. Similar to the protonated structures of anti-**5,5′-BBIm**, all p*K*_a_^−^ values at approximately 17.4 are found similar to each other and to p*K*_a_^−^ of 2-PhBIm (17.50). The relative energies of deprotonated structures **p** were found to be similar to each other, which in turn has little effect on p*K*_a_^2−^ at approximately 18.3.

Structural comparison of different protonation states of anti-**5,5′-BBIm** is made in Table 4. For the neutral state, there is very little difference in bond length (1.486 Å) or dihedral angle (44°) for neutral structures **n**. Solvation uniformly reduces the dihedral angle by 4°, something not seen in the structures studied so far. For charged states **m** and **p**, the bond lengths decrease slightly for both cases by approximately 0.004 Å. For the protonated molecules, contraction has been consistently observed, but for deprotonated molecules, this is the first case to be observed out of those that have been studied so far. For biprotonated (deprotonated) structures, the bond length between the benzimidazole units increases due to charge repulsion, but the effect is less pronounced compared to **2,5′-BBIm** and **2,2′-BBIm,** which suggests that the charge is localized on the imidazole moiety rather than benzimidazole as a whole. The effect of solvation on structure is less pronounced when compared to other structures. For structure **k**, the dihedral angle decreases by 6°, but for other structures the differences in bond lengths and dihedral angles are not significant.

Figure 11 and Figure 12 show p*K*_a_^2+^ and p*K*_a_^+^ values of syn-**5,5′-BBIm**, respectively. Although in terms of molecular shape, syn-**5,5′-BBIm** and anti-**5,5′BBIm** are very different, the effect on p*K*_a_^2+^ and p*K*_a_^+^ values are too minor to be discussed. Hence, the same arguments can be applied for syn-**5,5′-BBIm** that was applied in anti-**5,5′-BBIm**, where biphenyl moiety prevents any interaction with the protonated structures. Similarly, p*K*_a_^−^ and p*K*_a_^2−^ values shown in Figure 13 and Figure 14 are similar to those found in anti-**5,5′-BBIm** structures, and the same arguments may be applied for these values as well. The pattern in variation of bond lengths and dihedral angles show very little difference compared to anti-**5,5′-BBIm** (Table 5).

## 3. Methodology

### 3.1. Methodology of Acid Dissociation Constant Calculation

Equation (1) and thermodynamic cycle as shown in Scheme 1 was used for all p*K*_a_ calculations. As mentioned in the previous section, there are several factors that influence the accuracy of p*K*_a_ value. In general, we aim to follow the method presented by Psciuk et al. [27,34], where the p*K*_a_ and reduction potential of guanine, which is structurally similar to our molecule of interest, were calculated. $G^{o}\left(H_{\left(g\right)}^{+}\right)$ was taken as 6.28 kcal/mol, from Sackur-Tetrode equation [39]. For ${\Delta G}_{\mathrm{solv}}^{*}\left(H^{+}\right)$, we used the value −265.9 kcal/mol. The calculations for solvated state were completed using SMD, and molecules were optimized in the solvated state. Multiple protonated and deprotonated states were calculated in this study, resulting in multiple p*K*_a_ values for a single molecule. For example, **2,5′-BBIm** has two imidazole rings, both of which can be protonated and deprotonated. In the interest of simplicity, conformation is ignored in this description. Five different protonation levels are possible, starting from a fully deprotonated structure, single deprotonated structure, neutral structure, single protonated structure, and fully protonated structure. This gives rise to four p*K*_a_ values with notation p*K*_a_^n^, where n is the largest charge number of all the structure involved in proton dissociation. In the above case, there are p*K*_a_^2+^, p*K*_a_^+^, p*K*_a_^−^, and p*K*_a_^2−^. This leaves the term “p*K*_a_” free to use as a general term in discussions.

### 3.2. Computational Details

All the structures used for all p*K*_a_ calculations were optimized by DFT calculation using the long-range and dispersion-corrected ωB97X-D functional [40] and 6-311+G(d,p) split-valence triple-zeta basis set [41,42] with diffuse function. In this calculation, the int = ultrafine option was chosen for integration grids and the solvent effect of water was taken into account by the Polarizable Continuum Model (PCM) using the integral equation formalism (IEFPCM) [43] with radii and non-electrostatic terms for SMD solvation model [31]. The geometry optimization was checked by the following convergence criteria: i.e., the cutoff value of the maximum component of the force is 0.00045 harterees/bohr, that of the rot-mean-square of the forces is 0.0003 harterees/bohr, that of the maximum displacement for the next optimization step is 0.0018 bohr, and the root-mean-square of the displacement for the next optimization step is 0.0012 bohr. The frequency calculation was carried out using the optimized geometry, and the geometry was validated by lack of imaginary frequency. HOMO energy was also checked, in particular anionic species to ensure all electrons are bound to the molecule. The thermal correction of Gibbs free energy to the DFT total electronic energy was calculated using the translational, rotational, and vibrational motion components at 298.15 K and 1 atm based on statistical mechanics. All electronic structure calculations were performed using Gaussian 09 program package [44].

## 4. Conclusions

The p*K*_a_ of **2,5′-BBIm**, **2,2′-BBIm**, and **5,5′-BBIm** have been calculated using the ωB97X-D/6-311+G(d,p) level of theory and SMD solvation model. p*K*_a_ at several levels of protonation were investigated. **2,2′-BBIm** was found to be the most acidic with p*K*_a_^2+^ value of −2.67 and most acidic p*K*_a_^+^ value of 3.23. **2,5′-BBIm** was found to be second most acidic with the most acidic p*K*_a_^2+^ value of 3.57 and p*K*_a_^+^ value of 5.23. The most acidic p*K*_a_^2+^ and p*K*_a_^+^ values were 5.52 and 5.91, respectively. 

For **2,5′-BBIm**, there was a preference in protonation and deprotonation of imidazole. The terminal benzimidazole was energetically more favorable to be protonated, whereas the other benzimidazole was energetically more favorable to be deprotonated. For **2,2′-BBIm**, while no different conformation exists for protonated (deprotonated) structures, there were two conformations for neutral structures (**h1**, **h2**). **h1** was found to be more stable than **h2**, giving this conformation more acidic p*K*_a_^+^ value (3.23), and more basic p*K*_a_^−^ value (15.09). This molecule had the greatest structural change when protonated (deprotonated), due to large interaction between the two imidazole rings. On the other hand, **5,5′-BBIm** was least influenced by the protonation (deprotonation) position or state, giving consistently similar p*K*_a_^+^ and p*K*_a_^−^ value similar to **2-PhBIm**. As such, this molecule could be approximated as two independent **2-PhBIm** molecule.

In terms of application in use as polymer electrolyte membrane, an acidic molecule is desirable as such a molecule would be less prone to forming salt pair with phosphoric acid and more likely to encourage deprotonation and, therefore, proton transport. Thus, from the acid dissociation constant perspective, this structure maybe implemented in the polymer chain.

## Figures and Tables

**Table 1:** Bond length and dihedral angle between the benzimidazole and phenyl group of 2-phenylbenzimidazole (**2-PhBIm**) and their ionic counterparts both in the gas phase and aqueous phase at the ωB97X-D/6-311+G(d,p) level of theory.

Molecule	Gas Phase		Aqueous Phase	
	Bond Length (Å)	Dihedral Angle (°)	Bond Length (Å)	Dihedral Angle (°)
**2-PhBIm^+^**	1.455	33.90	1.458	24.19
**2-PhBIm**	1.469	17.21	1.469	20.73
**2-PhBIm^−^**	1.475	0.01	1.478	16.35

**Table 2:** The bond length and dihedral angle between the two benzimidazole repeating units of **2,5′-BBIm** and their ionic counterparts both in the gas phase and aqueous phase at the ωB97X-D/6-311+G(d,p) level of theory.

Molecule	Gas Phase		Aqueous Phase	
	Bond Length (Å)	Dihedral Angle (°)	Bond Length (Å)	Dihedral Angle (°)
**a**	1.467	42.97	1.459	27.72
**b1**	1.447	30.96	1.454	25.58
**b2**	1.467	16.34	1.469	24.58
**b3**	1.449	30.27	1.455	26.11
**b4**	1.468	7.84	1.470	24.55
**c1**	1.468	21.92	1.469	18.28
**c2**	1.469	15.84	1.469	24.09
**c3**	1.469	22.11	1.469	22.62
**c4**	1.468	17.19	1.469	23.14
**d1**	1.459	13.38	1.466	11.48
**d2**	1.477	0.02	1.480	8.80
**d3**	1.459	19.12	1.467	16.56
**d4**	1.474	0.07	1.479	8.07
**e**	1.483	0.00	1.480	9.05

**Table 3:** Bond length and dihedral angle between the two benzimidazole repeating units of 2,2′-bibenzo[d]imidazole and their ionic counterparts both in the gas phase and aqueous phase at the ωB97X-D/6-311+G(d,p) level of theory.

Molecule	Gas Phase		Aqueous Phase	
	Bond Length (Å)	Dihedral Angle (°)	Bond Length (Å)	Dihedral Angle (°)
**f**	1.457	49.64	1.450	7.76
**g**	1.441	0.01	1.447	4.61
**h1**	1.451	0.00	1.452	0.00
**h2**	1.463	35.57	1.459	18.80
**i**	1.462	0.01	1.461	0.04
**j**	1.486	35.29	1.475	20.59

**Table 4:** Bond length and dihedral angle between the two benzimidazole repeating units of anti-5,5′-bibenzo[d]imidazole in straight conformation and their ionic counterparts, both in the gas phase and aqueous phase at the ωB97X-D/6-311+G(d,p) level of theory.

Molecule	Gas Phase		Aqueous Phase	
	Bond Length (Å)	Dihedral Angle (°)	Bond Length (Å)	Dihedral Angle (°)
**k**	1.488	47.49	1.487	41.65
**m1**	1.481	42.77	1.486	40.63
**m2**	1.482	42.60	1.487	40.65
**n1**	1.485	43.96	1.487	39.73
**n2**	1.486	44.36	1.488	40.02
**n3**	1.487	43.61	1.488	40.26
**p1**	1.481	38.68	1.488	38.42
**p2**	1.483	39.42	1.489	39.11
**q**	1.492	40.89	1.490	38.61

**Table 5:** Bond length and dihedral angle between the two benzimidazole repeating units of 5,5′-bibenzo[d]imidazole in bent conformation and their ionic counterparts both in the gas phase and aqueous phase at the ωB97X-D/6-311+G(d,p) level of theory.

Molecule	Gas Phase		Aqueous Phase	
	Bond Length (Å)	Dihedral Angle (°)	Bond Length (Å)	Dihedral Angle (°)
**r**	1.489	50.64	1.486	40.99
**s1**	1.482	41.74	1.487	40.62
**s2**	1.481	43.87	1.486	40.16
**t1**	1.487	44.14	1.488	42.05
**t2**	1.486	43.59	1.487	40.92
**t3**	1.485	44.99	1.487	39.98
**u1**	1.484	40.41	1.488	39.63
**u2**	1.481	38.36	1.487	38.56
**v**	1.492	42.87	1.489	38.26

## Data Availability

Not applicable.
